# Effects of different Chinese traditional exercises on mental health during the COVID-19 pandemic: a systematic review and meta-analysis

**DOI:** 10.3389/fpubh.2024.1420035

**Published:** 2024-07-31

**Authors:** Shiqing Zhang, Min Liu, Lijin Zhao

**Affiliations:** Physical Education Institute, Shanxi University, Taiyuan, Shanxi, China

**Keywords:** Chinese traditional exercises, mental health, COVID-19, anxiety, depression

## Abstract

**Introduction:**

As the COVID-19 outbreak escalated into a global pandemic, there was a significant surge in mental health issues worldwide. Over the past three decades, traditional Chinese exercises (TCEs) has gained increasing recognition for its ability to regulate mental well-being. The aim of this study (PROSPERO CRD42024516002) was to comprehensively assess and carry out a meta-analysis on the impact of traditional Chinese exercise on personal mental well-being amidst the COVID-19 pandemic.

**Methods:**

Literature with publication dates from 2020 to 2023 was searched in four databases, including CNKI, Wanfang, Pubmed and Web of science. The literature was selected layer by layer according to the PRIMA guidelines, and then the quality of the included literature was assessed using the Cochrane Risk assessment tool.

**Results:**

A total of 174 articles were screened, and 10 studies met the criteria and were included in the study. The results showed that TCEs had a positive effect on anxiety symptoms [SMD = −1.68, *I^2^* = 98.40, 95%CI = (−2.80, −0.56), *p* = 0.00] and depressive symptoms [SMD = -1.23, *I^2^* = 89.23, 95%CI = (−1.87, −0.58), *p* = 0.00]. The data of subgroup analysis showed that Baduanjin exercise had the best effect on reducing anxiety [SMD = −2.29, *I^2^* = 98.3, 95%CI = (−3.69, −0.89), *p* < 0.000]. Individuals who practiced TCEs less than or equal to 30 min each time had the best effect on anxiety [SMD = −2.06, *I^2^* = 96.2%, *p* < 0.000] and depression [SMD = −1.90, *I^2^* = 68.4, 95%CI = (−2.25, −1.55), *p* = 0.042]. Individuals who trained TCEs more than 7 times a week were most likely to reduce symptoms of anxiety [SMD = −4.30, *I^2^* = 92.6, 95%CI = (−6.78, −1.81), *p* < 0.000] and depression [SMD = −2.39, *I^2^* = 0.0, 95%CI = (−2.91, −1.87), *p* = 0.625]. It is worth noting that TCEs had the most significant effect on the improvement of depression in people aged 30–50 years [SMD = −1.58, *I^2^* = 89.0, 95%CI = (−3.05, −0.10), *p* = 0.003].

**Conclusion:**

During the global pandemic, traditional Chinese sports have shown a positive and significant impact on reducing symptoms of anxiety and depression, and have played a significant role in improving mental health problems.

**Systematic review registration:** PROSPERO, identifier CRD42024516002, https://www.crd.york.ac.uk/prospero/display_record.php?ID=CRD42024516002.

## 1 Introduction

In December 2019, a novel coronavirus struck Wuhan, China, causing a spike in pneumonia clusters. The rapid spread of the virus cluster across the globe is staggering. Given its serious harm and widespread spread, the World Health Organization (WHO) took the important decision on March 11, 2020, to officially declare Severe Acute Respiratory syndrome Coronavirus 2 (SARS-CoV-2) a global pandemic (1). In the face of a global health crisis, people are not only facing physical threats, but also experiencing enormous psychological stress and emotional distress. In terms of physiology, the most common clinical symptoms of COVID-19 infection are fever, cough, sore throat, diarrhea, vomiting, etc., and the virus not only affects the lungs, but also affects tissues such as the heart and kidneys (2), especially people with underlying medical conditions, who may have more severe symptoms (3). Psychologically, students have been forced to switch to online learning due to lockdowns and self-isolation, middle-aged people are working from home or at risk of losing their jobs, and the older adult are not fully aware of prevention and control information (4), which has caused them to bear greater stress and anxiety during the epidemic. Individuals’ fear of the unknown, prolonged lockdowns, and limited scientific information exacerbate mental health problems such as anxiety, depression, fear, and bipolar depression in these groups (4–8). In-depth studies have revealed that up to 54% of the general population is experiencing significant psychological stress during this global health crisis (9), with a 27.6% surge in the prevalence of depression and a 25.6 percent increase in the prevalence of anxiety disorders (10–13). This trend shows that more and more people are suffering from severe challenges of mental illness during the pandemic. Focusing further on China, the initial outbreak of the pandemic, it is worrying that 16.5% of respondents showed moderate to severe depressive symptoms, while 28.8% showed moderate to severe anxiety symptoms (14). These data once again confirm the huge impact of the epidemic on people’s mental health, especially in countries and regions where the epidemic situation is severe.

Medication and psychological counseling are the most common interventions for mental health problems, but drug treatment may have certain side effects on the body, and psychological counseling has a limited duration. More scholars have shifted their research direction to exercise for mental health problems, and the results have shown that exercise is beneficial to mental health (15–17), and doing exercise is negatively associated with having mental health problems such as anxiety and depression (18, 19). Traditional Chinese Exercises (TCEs), such as martial arts and qigong, have attracted attention for their unique forms of exercise and health preservation concepts. This type of exercise is characterized by peaceful and smooth movements combined with the harmony of body and mind, focusing on the coordination and unity of mind and movement, and has a unique health care effect. They not only enhance cardiopulmonary function (20, 21) and exercise capacity (22), but are also effective in alleviating negative emotions (23–25). In the context of today’s fast-paced and high-stress life, TCEs provide an effective intervention for the rehabilitation of chronic diseases (26). Over the past three decades, TCEs have gained international recognition for health promotion (27), and a growing body of research has confirmed that TCE is an effective means of intervening in mental health. In the context of the novel coronavirus (COVID-19) epidemic as a public health emergency, this conclusion still holds true. For example, one study confirmed that Baduanjin helped reduce perceived anxiety associated with COVID-19 during the pandemic and could improve the mental health of college students (28). Practicing Tai Chi can improve cognitive status and mental health (29). In addition, studies have shown that traditional Chinese sports (e.g., Chinese martial arts, tai chi, and qigong) have improved mental health during the COVID-19 pandemic (30).

However, when exploring the effects of traditional Chinese sports on mental health, we face a significant challenge: the sample size for this specific context is relatively limited, and there are significant differences in the effects of different traditional exercises (such as Baduanjin, Yijinjing, Wushu, etc.) on mental health interventions. In order to improve the credibility and representativeness of the study, it is important to expand the sample size. How do different TCEs differ in their impact on individual mental health? Are there differences in the impact of TCEs on individual mental health among different age groups? Compared with normal times, can TCEs as an intervention be able to alleviate mental health problems such as anxiety and depression caused by the epidemic more significantly?

In summary, this article conducts a detailed systematic review and meta-analysis to further explore and address the specific impact of TCEs on mental health during the pandemic. Through this study, we have obtained more concrete and accurate conclusions, which effectively fills the research gap in this field. This will not only help us to deeply understand the application value of TCEs in the field of mental health, encourage more people to pay attention to and participate in TCEs, so as to promote the mental health and well-being of individuals and communities, but also provide powerful psychological intervention strategies for coping with emergencies such as epidemics.

## 2 Method

This study has been registered with PROSPERO with registration number CRD42024516002. Furthermore, the requirements outlined in the Priority Reporting Items for Systematic Reviews and Meta-Analyses (PRISMA Guidelines) (31) were conducted in order to improve the scientific and comparability of systematic reviews.

### 2.1 Search strategy

Two independent researchers conducted an extensive search for randomized controlled trials in four databases, CNKI, Wanfang, Pubmed, and Web of Science, and the language restrictions in the foreign language databases were English, and all the literature searched were published studies, so there was no ethical approval and patient consent was required. In view of the outbreak of the novel coronavirus pneumonia epidemic at the end of 2019 and the major adjustment of epidemic prevention and control policies worldwide in January 2023, the literature of this meta-analysis was collected from the research results published between 2020 to 2023. Chinese search terms include: “CoV-19”OR“SARS-CoV-2,”“mental health,”“Tai-ji”OR“Baduan jin”OR“liuzijue”OR“Qigong,” etc. Searches are conducted using subject headings and free words, more information about search strategies can be found in the Supplementary materials.

### 2.2 Inclusion and exclusion criteria

The inclusion criteria were established using the PICOS approach (study participants, interventions, comparative measures, findings, and study design). A study is eligible for inclusion if it meets the following criteria: (1) Study subjects: any group during the COVID-19 epidemic, including people with COVID-19, convalescent patients, and healthy people. The lack of population restrictions ensured the comprehensiveness of the study’s analysis. (2) Interventions: Traditional Chinese sports were used as interventions, including Baduanjin, Yijinjing and other projects. There are no specific restrictions on the number, frequency, duration and intensity of interventions. (3) Comparator measures: The control group did not receive the intervention or only received usual care or other sports programs. (4) Research results: Based on the mental health level of the research subjects, this article focuses on two factors: anxiety and depression. No specific restrictions on mental health testing (5) Study design: including peer-reviewed randomized controlled trials.

The exclusion criteria were as follows: (1) reviews and letters, conferences, etc., (2) low-quality articles or dissertations, (3) articles for which full text or data could not be extracted, (4), mismatched outcomes, (5) animal experiments, and (6) non-randomized controlled trials.

### 2.3 Study selection and data extraction

All retrieved documents were imported into EndNote21 software and deduplicated. Two researchers (ZSQ and ZLJ) independently combed and screened the literature by reading the title, abstract and full text. If there is a disagreement on article inclusion, the final decision is made in consultation with the third investigator. The information of the included literature was extracted using Excel software, including authors, year of publication, demographic characteristics (age, gender, presence or absence of diseases), specific intervention and control programs (measures, frequency, duration and duration), measurement tools and outcome indicators.

### 2.4 Quality appraisal

The Cochrane Bias risk assessment tool was used to assess the quality of the included studies. Using the bias table of RevMan 5.4 software, we comprehensively considered seven key dimensions: random sequence generation, assignment hiding, participant blindness, outcome variable evaluation blindness, outcome data missing, selective reporting, and other biases. The quality assessment was carried out independently by two researchers (ZSQ and ZLJ) to ensure the impartiality and accuracy of the assessment. During the evaluation process, the two researchers strictly followed the evaluation criteria, and each study was carefully reviewed. In case of disagreement at any link, a consensus is reached through in-depth discussion and consultation with the third researcher (LM) to ensure the objectivity and reliability of the evaluation results.

### 2.5 Data synthesis and analysis

In this study, Stata 17.0 software was used for in-depth data analysis. In the process of data analysis, we mainly focus on two variables, anxiety and depression, and treat them as continuous variables. To more accurately assess the difference between the experimental and control groups, we used the standard mean difference (SMD) and 95% confidence interval (95% CI) as a difference scale for the size of the combined difference. Statistically, we used Chi-square tests to assess heterogeneity across studies. When the *I^2^* value is less than 50%, the heterogeneity among the included studies is low and the data are relatively consistent. When the value of *I^2^* is greater than 50%, it means that there is high heterogeneity between studies, and there may be some differences in data results. Based on the heterogeneity of the evaluation results, we selected the appropriate statistical model. When there is high heterogeneity, in order to reflect the real situation more accurately, we choose the random effects model. In contrast, when the heterogeneity is low, we use the fixed-effect model. In addition, we set strict significance levels. When the *p*-value was less than 0.05, we considered that there was a significant difference between the experimental group and the control group, thus determining that the results of the meta-analysis were statistically significant.

## 3 Results

### 3.1 Study selection

**Figure 1:**
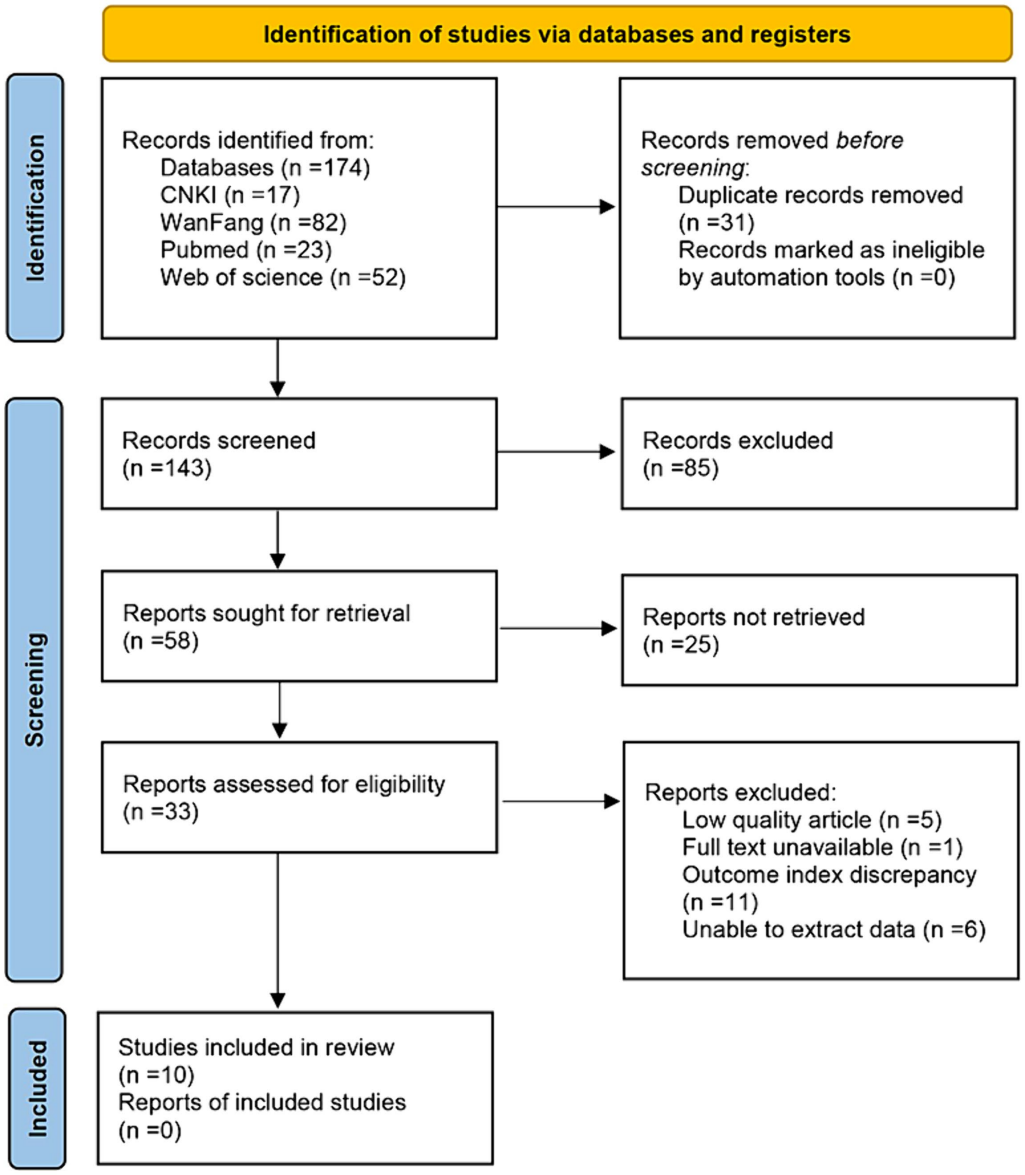
PRISMA 2020 flow diagram.

Initially, we systematically searched CNKI, WanFang, Pubmed, and Web of Science databases, and collected a total of 174 relevant articles. To ensure the rigor of the studies, we first removed 31 duplicates and narrowed the total number to 143. Then, by carefully reviewing the titles and abstracts of these literatures, 85 articles that were not closely related to the research topic were excluded. Finally, after in-depth reading and analysis of the remaining literature, we identified 10 documents that fully met the requirements of this study. Based on these 10 articles, we conducted a detailed meta-analysis with a view to drawing accurate and reliable conclusions (see Figure 1).

### 3.2 Study characteristics

A total of 10 randomized controlled trials were included in the literature for screening and exclusion. Table 1 shows the main characteristics of the included literature. The time span of the included studies was from 2020 to 2023, with a total sample size of 1,165 participants (581 in the intervention group and 584 in the control group).

**Table 1:** Characteristics of studies included in this meta-analysis.

Author	Year	Sample size	Age (*M* ± SD)	Sex (Male, *n*, %)	Subject	Intervention	Duration of a single intervention	Duration of Intervention	Outcome measures	Outcome
Chen et al. (32) RCT	2020	EG:14 CG:15	EG: 67.6 ± 11.2 CG: 68.5 ± 10.8	EG: 7(50%) CG: 6(40%)	coVID-19 patients	EG:Baduanjin CG:Routine care	Twice a day, 20-30 min each time	3 weeks	SAS SDS	anxiety depression
Han et al. (33) RCT	2022	EG:46 CG:46	EG:62.86 ± 5.16 CG:62.83 ± 5.14	EG: 26(56.52%) CG: 25(54.35%)	Patients with coronary heart disease	EG:Baduanjin CG:Routine care	Five times a week,30 min each time	12 weeks	HAMA、HAMD	anxiety depression
Li et al. (28) RCT	2022	EG:195 CG:192	20–30 (Range)	197(50.9%)	College students	EG:Baduanjin CG:No intervention	At least 4 times a week, 45 min each time	12 week	CAS	anxiety
Ma et al. (34) RCT	2023	EG:35 CG:35	EG: 36.82 ± 11.68 CG: 40.29 ± 8.66	EG: 14(40%) CG: 17(48.57%)	coVID-19 patients	EG:Baduanjin CG:Routine care	Twice a day, 15 min each time	10 days	PHQ-9	depression
Solianik et al. (35) RCT	2021	EG:15 CG:15	60–78 (Range)	EG:2(13.33%) CG:2(13.33%)	older adult	EG:Tai Chi CG:No intervention	a biweekly 60-min	10 weeks	HADS	anxiety depression
Wang et al. (36) RCT	2021	EG:100 CG:100	19–21 (Range)	/	healthy people	EG:Baduanjin CG:No intervention	Three times a week,90 min each time	18 weeks	SCL-90	anxiety depression
Wu et al. (37) RCT	2020	EG:58 CG:57	51.30 ± 8.45	EG:0(0%) CG:0(0%)	College students	EG:Baduanjin CG:Routine care	Twice a day, 30-60 min each time	16 days	SAS	anxiety
Xing et al. (38) RCT	2023	EG:74 CG:80	EG: 44.0 ± 11.3 CG: 42.5 ± 12.7	EG:31(41.89%) CG:33(41.25%)	coVID-19 patients	EG: Yijinjing CG:Routine care	Every day,20 min each time	EG:7.4 ± 2.6 days CG:7.5 ± 2.5 days	HAMA	anxiety
Zhang and Rao (39) RCT	2020	EG:14 CG:14	EG: 49.87 ± 4.23 CG: 49.89 ± 4.22	EG:8(57.14%) CG:9(60%)	coVID-19 patients	EG: Yijinjing CG:No intervention	Six times a week,60 min each time	8 weeks	SAS SDS	anxiety depression
Zhang (40) RCT	2021	EG:30 CG:30	/	/	College students	EG:Traditional fitness exercises CG:No intervention	Once a day, 60 min each time	1 week	SAS SDS	anxiety depression

EG experimental group. CG control group. /No data available.

In terms of exercise interventions, six interventions were Baduanjin (60 percent) (28, 32–34, 36, 37), two were Yijinjing (20 percent) (38, 39), and the remaining two were Tai Chi (35) and traditional fitness exercises (40). The age of the population is broadly divided into three categories, including 30 years of age or less (28, 36, 40), 30 to 50 years of age (34, 38, 39), and 50 years of age or older (32, 33, 35, 37). The duration of a single intervention also varies, ranging from <60 min (28, 32–34, 37, 38) to ≥60 min (35, 36, 39, 40). The frequency of interventions also varied widely, ranging from twice a day to once every 2 weeks (35). The intervention period ranged from 1 week to 18 weeks, with an average intervention period of 7.26 weeks.

Regarding intervention outcomes, nine studies (28, 32, 33, 35–40) assessed anxiety and seven studies (32–36, 39, 40) reported levels of depression. The assessment scales selected by the different studies differed, with SAS (32, 37, 39, 40), HAMA (33, 38), and CAS (28) for assessing anxiety, PHQ-9 (34), SDS (32, 39, 40), HAM (33), and the SCL-90 (36) and HADS (35) scales that measure anxiety and depression simultaneously.

### 3.3 Risk-of-bias assessment

**Figure 2:**
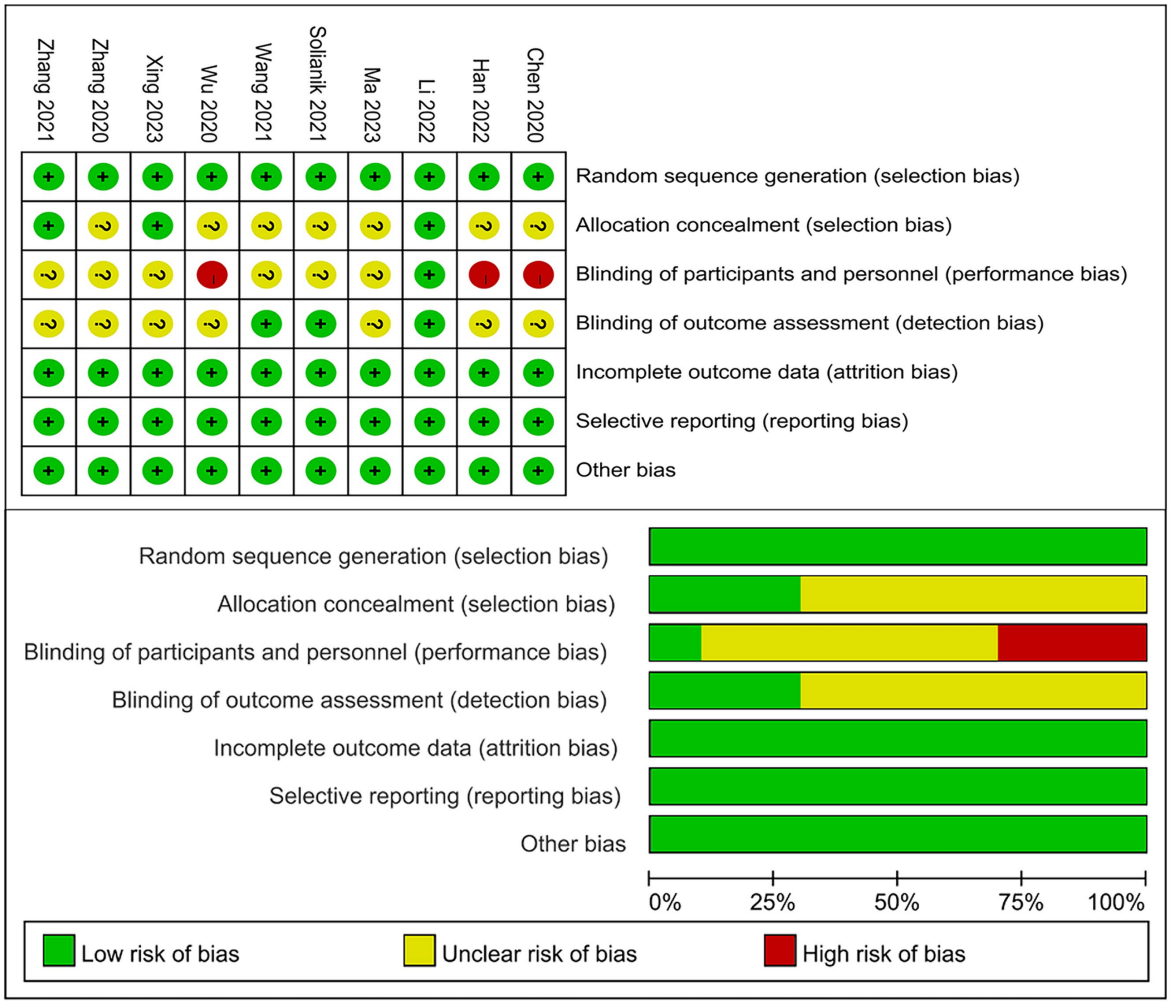
Risk-of-bias summary. Above: Risk of bias summary: review authors’ judgements about each risk of bias item for each included study. Below: Risk of bias graph: review authors’ judgments about each risk of bias item presented as percentage across all included studies.

Figure 2 summarizes the risk of bias. Overall, the risk of bias of the 10 trials included in the review was acceptable. Randomized sequences were adequately identified in all trials, full points were identified in three trials (30%), allocation was concealed in three trials (30%), outcome assessors were blinded in three trials, and selective reporting, incomplete outcome data, and other biases were absent for all items. As a result, the trials were rated as low risk of reporting bias. At the same time, the jadad scale developed by Alejandro Jadad-Bechara, which divided the literature into two grades: low quality (0–3 points) and high quality (4–7 points). The 10 articles included in this study were all high-quality literature, of which 40% were scored 4 and 5, and 10% were scored 6 and 7.

### 3.4 Results of meta-analysis

In the included trials, different trials used different mental health assessment tools to measure participants’ mental health during the pandemic. This review analyzed two aspects of participants’ anxiety and depression, and the results of the analysis of these two aspects are as follows.

#### 3.4.1 Anxiety

**Figure 3:**
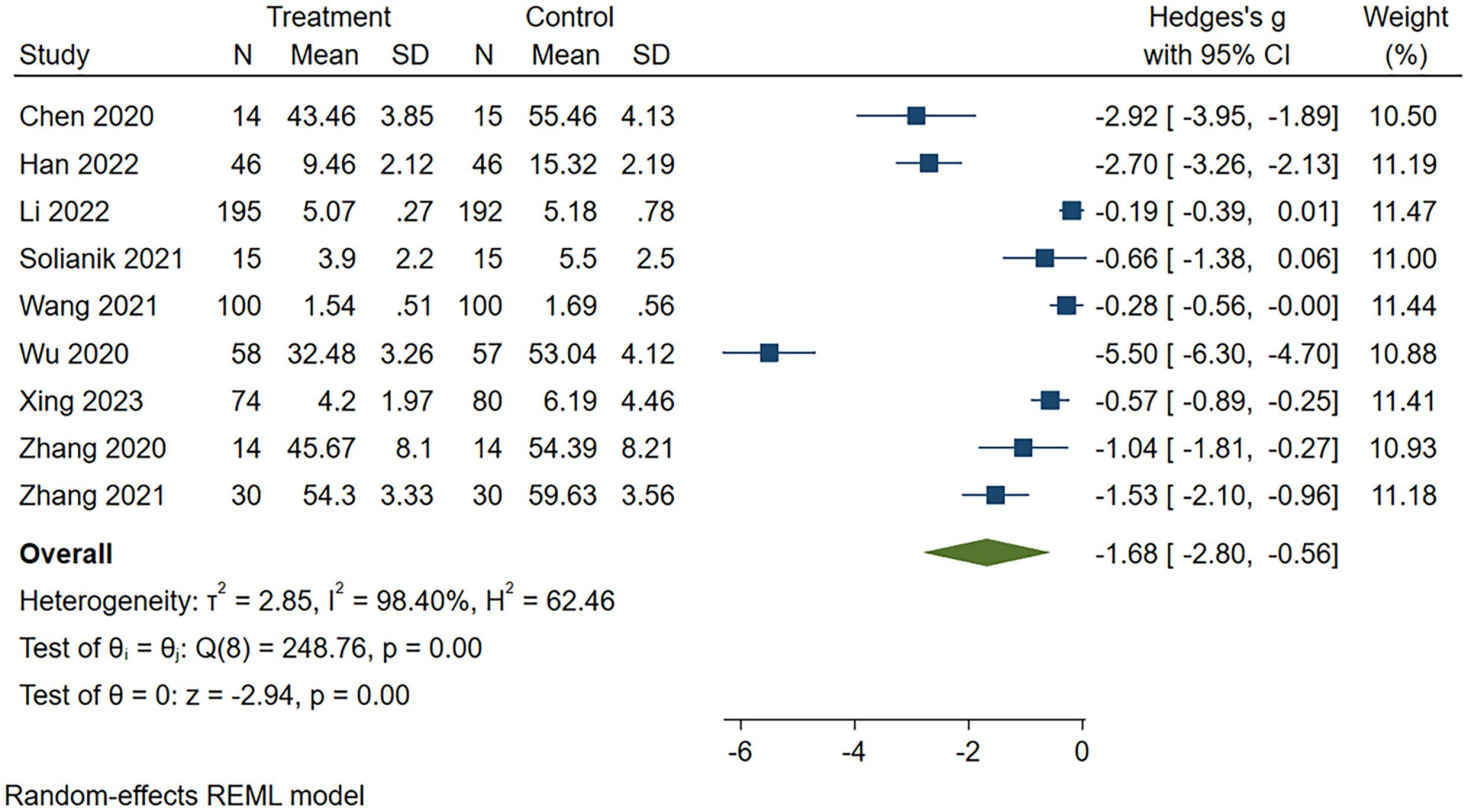
Forest plot for exercise on anxiety. CI, confidence interval.

Of the 10 included articles, 9 (28, 32, 33, 35–40) documented the effects of traditional Chinese sports on anxiety. The results showed a total sample size of 1,095 participants (546 in the intervention group and 549 in the control group), and there was substantial evidence that TCEs had a statistically significant positive effect on reducing anxiety compared with the control group [SMD = −1.68, 95% CI = (−2.80 to −0.56), *p* = 0.00; see Figure 3]. Given the heterogeneity observed in this study (*I^2^* = 98.40%, *p* = 0.00, see Figure 3), a random-effects model was selected and further exploratory analyses were performed by subgroup analyses to identify potential sources of heterogeneity.

#### 3.4.2 Depression

**Figure 4:**
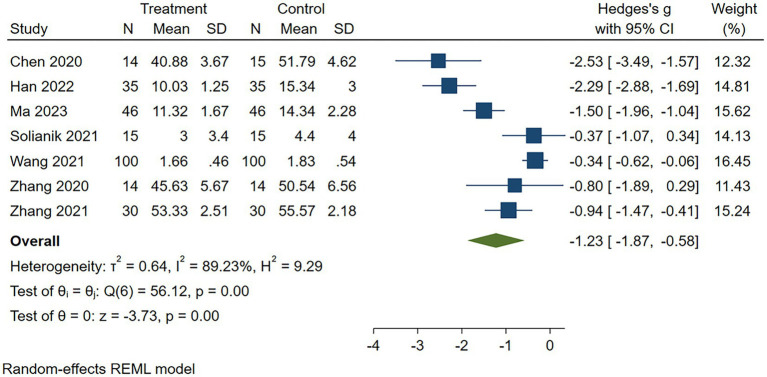
Forest plot for exercise on depression. CI, confidence interval.

Of the 10 included studies, 7 reported on the effect of traditional Chinese exercise on depression (32–36, 39, 40), involving a total of 509 participants (254 in the intervention group and 255 in the control group). Through detailed data analysis, we found a high heterogeneity between these studies (*I^2^* = 89.23%, *p* = 0.00, see Figure 4). Given this high degree of heterogeneity, we chose a random-effects model for analysis. The results of the analysis showed that there was a significant difference in the level of depression between the traditional exercise intervention group and the control group, and individual practice of TCEs had a positive effect on reducing depressive symptoms [SMD = -1.23, 95%CI = (−1.87, −0.58); see Figure 4].

### 3.5 Sensitivity analysis

**Figure 5:**
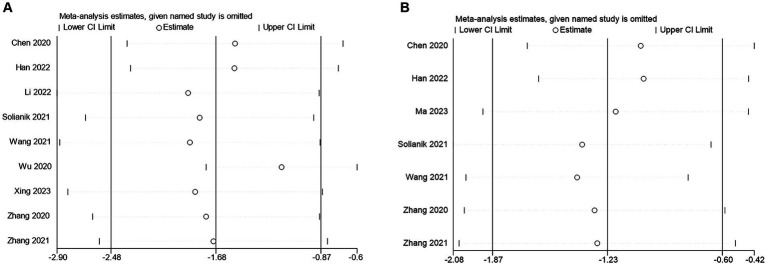
Sensitivity analysis of **(A)** anxiety **(B)** depression.

Sensitivity analysis is an important statistical method that allows us to assess the impact of each study on the overall results by systematically deleting individual studies, thus testing the robustness and reliability of the results. In this meta-analysis of TCEs interventions for anxiety and depression, we performed sensitivity analyses to gain a deeper understanding of the effects of TCEs interventions (Figure 5).

First, a meta-analysis of TCEs interventions on individual anxiety yielded statistically significant results. The odds ratio (OR) is −1.68 and the 95% confidence interval (CI) is −2.48 to −0.87. This means that TCE intervention has a significant relief effect on anxiety symptoms. In the sensitivity analysis, we found that the intervention effect of TCEs for anxiety disorders remained significant even after excluding individual studies, showing the robustness of the results.

Similarly, meta-analyses of TCEs on individual depression showed statistically significant results. The OR was −1.25, with a 95%CI of −1.91 to −0.59. This suggests that TCEs have a significant effect on reducing depressive symptoms in individuals. This is further supported by the results of the sensitivity analysis, which showed that the intervention effect of TCE for depression remained significant even when certain studies are excluded from the intervention of TCEs on individual depression.

### 3.6 Subgroup analysis

The results showed high heterogeneity in both anxiety and depression, so subgroup analyses were performed for both. It was divided into five parts, including interventions, duration of a single intervention, intervention frequency, intervention period, and participant age. Within these subgroups, factors exhibited varying degrees of heterogeneity. Subgroup analyses of anxiety and depression are described below.

#### 3.6.1 Anxiety

**Figure 6:**
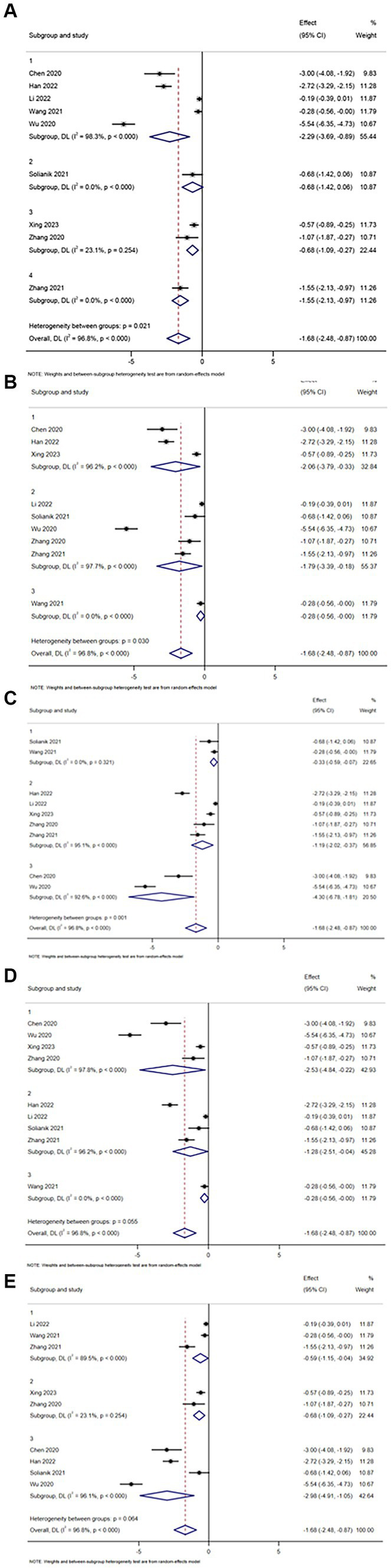
Anxiety subgroup analysis of **(A)** interventions, **(B)** single intervention duration, **(C)** exercise frequency, **(D)** intervention period, and **(E)** age of the subject.

Regarding interventions – Baduanjin (Group 1), Tai Chi (Group 2), Yijinjing (Group 3) and Chinese Fitness Exercises (Group 4). The intervention effect on anxiety disorders was as follows: Baduanjin (SMD = −2.29, *I^2^* = 98.3%, *p* < 0.000), Taiji (SMD = −0.68, *I^2^* = 0.0%, *p* < 0.000), Yijinjing (SMD = −0.68, *I^2^* = 23.1%, *p* < 0.000), traditional fitness exercises (SMD = −1.55, *I^2^ =* 0.0%, *p* < 0.000), Yijinjing (SMD = −0.68, *I^2^* = 23.1%, *p* < 0.000). Except for the Yijinjing group, which was not statistically significant (*p* = 0.254), there were statistically significant differences between the Baduanjin group, the Taiji group and the traditional fitness exercise group (*p* = 0.05), and it is worth noting that the Baduanjin group had the most significant effect on reducing anxiety (refer to Figure 6A).

Regarding the duration of a single intervention – less than or equal to 30 min (Group 1), 30–60 min (Group 2), greater than 60 min (Group 3). The intervention effect on anxiety was less than or equal to 30 min group (SMD = −2.06, *I^2^* = 96.2%, *p* < 0.000), 30-60 min group (SMD = −1.79, *I^2^ =* 0.0%, *p* < 0.000), and greater than 60 min group (SMD = −0.28, *I^2^* = 0.0%, *p* < 0.000). The difference between the three groups (*p* < 0.000) was statistically significant (refer to Figure 6B).

Regarding the frequency of intervention – less than or equal to 3 times per week (Group 1), 5–7 times per week (Group 2) and more than 7 times per week (Group 3). The intervention effect of anxiety in each group was as follows: less than or equal to 3 times per week (SMD = −0.33, *I^2^* = 0.0%, *p* = 0.321), 5–7 times per week (SMD = −1.19, *I^2^* = 95.1%, *p* < 0.000) and more than 7 times per week (SMD = −4.30, *I^2^* = 92.6%, *p* < 0.000). There was a statistically significant difference between the groups 5–7 times per week and more than 7 times per week (*p* < 0.05), while there was no significant difference in the group less than or equal to 3 times per week (*p* = 0.321; refer to Figure 6C).

In subgroup analyses of anxiety, intervention duration (*p* = 0.055) and participant age (*p* = 0.064) had no significant effect on TCEs in reducing anxiety symptoms (refer to Figures 6D,E).

#### 3.6.2 Depression

**Figure 7:**
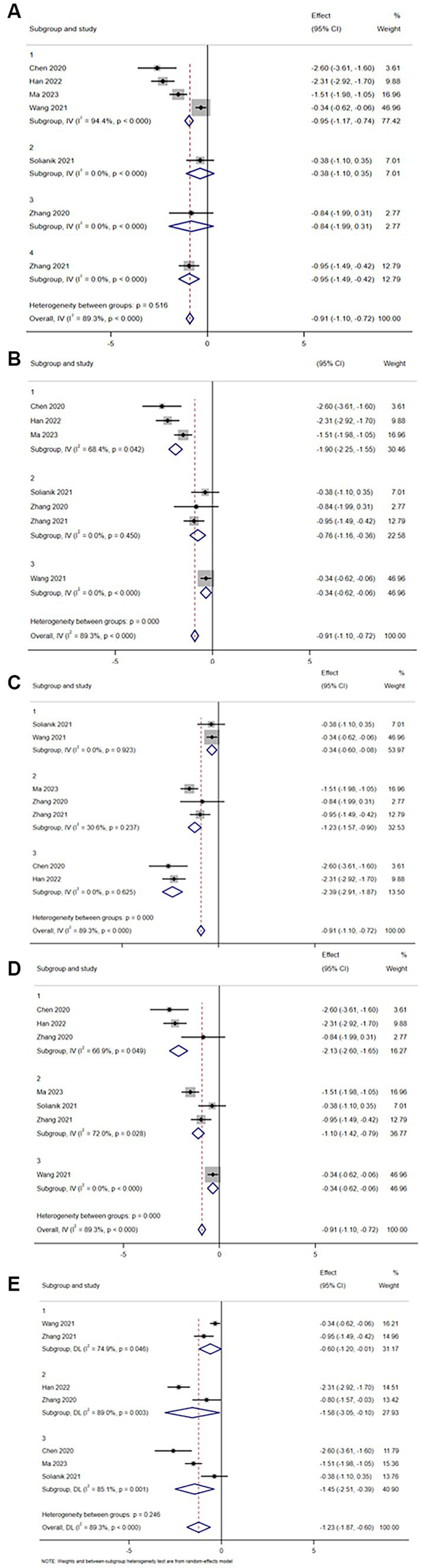
Depression subgroup analysis of **(A)** interventions, **(B)** single intervention duration, **(C)** exercise frequency, **(D)** intervention period, and **(E)** age of the subject.

In a subgroup analysis of depression, the intervention (*p* = 0.516) had no significant effect on TCEs in reducing anxiety symptoms (refer to Figure 7A).

Regarding the duration of a single intervention – less than or equal to 30 min (Group 1), 30–60 min (Group 2) and greater than 60 min (Group 3). The effects of TCEs on different single intervention durations were as follows: less than or equal to 30 min (SMD = −1.90, *I^2^* = 68.4%, *p* = 0.042), 30-60 min (SMD = −0.76, *I^2^* = 0.0%, *p* = 0.450) and greater than 60 min (SMD = −0.34, *I^2^* = 0.0%, *p* < 0.000). There was no statistically significant difference in Group2, but there was a statistically significant difference in the other two groups (*p* < 0.05; refer to Figure 7B).

Regarding the frequency of intervention – less than or equal to 3 times per week (Group 1), 5–7 times per week (Group 2) and more than 7 times per week (Group 3). The effect of the intervention on depression was as follows: less than or equal to 3 times a week (SMD = −0.34, *I^2^* = 0.0%, *p* = 0.923), 5–7 times a week (SMD = −1.23, *I^2^* = 30.6%, *p* = 0.237), and more than 7 times a week (SMD = −2.39, *I^2^* = 0.0%, *p* = 0.625). There was no significant difference between the three groups (*p* > 0.05; refer to Figure 7C).

Regarding the duration of the intervention – the duration of the intervention was 3 weeks or less (Group 1), 10–12 weeks (Group 2) and greater than 12 weeks (Group 3). The effects of the intervention on depression were as follows: the duration of the intervention cycle was less than or equal to 3 weeks (SMD = −2.13, *I^2^* = 66.9%, *p* = 0.049), 10–12 weeks (SMD = −1.10, *I^2^* = 72.0%, *p* = 0.028), and greater than 10 weeks (SMD = −0.34, *I^2^* = 0.0%, *p* < 0.000). The differences between the three groups were statistically significant (*p* < 0.05; refer to Figure 7D).

Regarding the age of participants – they were classified as 30 years or less (Group 1), 30–50 years (Group 2) and 50 years or older (Group 3). The intervention effect of TCEs on different age groups was as follows: less than or equal to 30 years old (SMD = −0.60, *I^2^* = 74.9%, *p* = 0.046), 30–50 years old (SMD = −1.58, *I^2^ =* 89.0%, *p* = 0.003), and over 50 years old (SMD = −1.45, *I^2^* = 85.1%, *p* = 0.001). The differences in the three groups were statistically significant (*p* < 0.05; refer to Figure 7E).

## 4 Discussion

### 4.1 Summarize the main results of the article

The sudden outbreak of the new crown pneumonia has caused fear and panic among people, and the prolonged isolation has caused people to have more negative emotions than before. TCEs have their unique psychological regulation effects, and are gradually becoming internationalized and recognized by more and more countries, but there is a lack of systematic evaluation and meta-analysis of traditional Chinese sports on mental health during the pandemic. Therefore, a total of 10 articles were selected for analysis in this meta-analysis to explore the impact of TCEs on mental health in the context of the pandemic from the perspectives of anxiety and depression. Research data suggests that TCEs during the pandemic have a positive effect on mental health improvements. We systematically assessed all the included studies, 10 of which were randomised controlled studies, read and extracted sample characteristics (including first author, date of publication, intervention design, age and gender, etc.), and conducted risk assessments, all of which were of high quality, which increased the credibility of this meta-analysis and made the conclusions more accurate. Due to the restrictions of the epidemic and other restrictions, the experiment could not be completely blinded, but the impact on the experimental results was weak. This is because previous studies have shown that there is a potential risk of bias even when the best methods are used (41). Therefore, the quality of the included literature cannot be denied because it is not blinded.

### 4.2 Analysis of the effects of exercise program intervention

Through the above data, we found that there are many factors that affect the effect of traditional Chinese exercise on mental health intervention. In terms of interventions, traditional Chinese sports have a long history, and each sport carries a different cultural heritage and historical accumulation, so that each of them has a unique effect in promoting mental health. Baduanjin, a mind–body exercise inspired by Taoist philosophy and Chinese medicine theory, guides and strengthens the flow of “qi” in the body, aiming to improve the physical and mental health of individuals in an all-round way (42–44). Taijiquan, as a treasure of traditional Chinese martial arts, is deeply rooted in the ancient Chinese doctrine of “yin and yang” and the philosophy of “tai chi” (45). During the exercise, the body maintains a half-squat posture and moves slowly, which not only stabilizes the lower plate, but also embodies the wisdom of yin and yang balance between movement and stillness. The Yijinjing is a comprehensive mind–body movement method that integrates form, qi, and spirit (46), including a variety of movements such as concentration, walking, sitting, lying, and uprightness, aiming to optimize the body’s physiological functions and mental state through a variety of movement exercises (47). Notably, studies have shown that Baduanjin has shown a particularly effective effect in the rehabilitation of people with insufficient physical activity and certain medical conditions (3, 48). In view of the significant reduction in the amount of exercise caused by people’s long-term stay at home and limited activity space during the global pandemic, and the fact that the research sample was mostly focused on patients with the new crown pneumonia epidemic, Baduanjin showed the most significant effect in alleviating the anxiety that prevailed during the pandemic due to its low intensity and high adaptability.

Another factor influencing the mental health effects of TCEs is the programme of interventions, with the frequency, timing and duration of individual interventions all being crucial considerations. Especially in the context of the COVID-19 pandemic, Pegg’s research highlights the importance of proper exercise planning: it is recommended to aim for at least 30 min of moderate physical activity per day, or at least 20 min of vigorous physical activity every other day. This guideline aims to balance the subtle relationship between the benefits of exercise and the additional fatigue and psychological stress that can be triggered by over-exercising (49).

Age has a significant impact on the effectiveness of traditional Chinese sports in promoting mental health. As individuals age, their ability to exercise decreases, and physical functions such as stride speed, horizontal span, and lack of flexibility and physical strength are barriers that limit the participation of older adult in high-intensity physical activity (50). For this reason, most older adult tend to prefer low-intensity, soft-moving, and inflexible activities when choosing exercise modalities (51). In this context, traditional Chinese sports, with their unique charm and adaptability, are deeply loved and respected by middle-aged and older adult groups. Therefore, traditional Chinese sports have shown particularly significant results in improving the mental health of middle-aged and older adult people. These sports not only meet the physical conditions of middle-aged and older adult people, but also contain rich cultural connotation and health wisdom, which can help relieve stress and improve emotional state, so as to comprehensively promote the mental health of middle-aged and older adult people.

A key factor influencing the effectiveness of traditional Chinese exercise in intervening in mental health is individual differences. These differences are rooted in the physiology inherent to individuals, resulting in different degrees of adaptation to movement patterns and resulting psychological responses (50). Under this framework, some individuals show a significant preference for long-term, high-intensity exercise, in which they may find a way to challenge themselves and release stress. On the other hand, the other group of people is more inclined to choose short-cycle, low-frequency forms of exercise to achieve harmony and balance between body and mind (41). This multi-faceted differentiation of exercise preferences is not only a vivid interpretation of individual differences, but also requires us to have a high degree of individualized perspective and refined consideration when designing exercise intervention strategies to fully meet the unique needs and potential of each participant.

### 4.3 How TCEs could be used to prevent mental health issues during pandemics or other stressful periods

During the COVID-19 pandemic or other stressful times, utilizing traditional Chinese exercises to prevent mental health issues is a positive and effective approach. First of all, it is necessary to gain an in-depth understanding of the multiple advantages of traditional Chinese sports: it not only strengthens the body, but also deeply reflects the concept of promoting the harmony and unity of body and mind, and the cultivation of both internal and external aspects. This type of exercise has shown significant effects on emotional regulation, stress relief, and mental health improvement, making it an exercise choice for people of all ages. Especially in the context of the ongoing global pandemic, traditional Chinese sports have allowed people to enjoy exercise at home or in a safe environment with their unique charm, effectively coping with the restrictions and challenges brought about by the epidemic (52).

Second, it’s crucial to create a personalized exercise plan. Set a reasonable exercise frequency and goal according to your physique, health status and interests. Following the guidance of experts such as Pegger, at least 30 min of moderate exercise per day, or 20 min of vigorous exercise every other day, is a scientific and feasible starting point (3). This arrangement helps to keep the body active while avoiding the physical and mental exhaustion caused by excessive exercise. In addition, we encourage the integration of exercise into daily life, form regular exercise habits, and try a variety of traditional Chinese sports forms to make sports more colorful and stimulate the motivation to continue to participate.

In addition, the combination of psychological counseling and education is also indispensable. In the face of additional psychological pressure, providing online and offline professional psychological counseling services has become an important way to alleviate psychological distress and prevent mental health problems. At the same time, it is necessary to popularize mental health education, enhance the public’s awareness and understanding of mental health, and teach effective methods to cope with stress and adjust emotions, so as to improve the psychological resilience of individuals.

Finally, make full use of Internet technology to create a convenient and efficient sports learning platform. Through online courses and video tutorials, people can easily master traditional Chinese sports skills and enjoy sports anytime and anywhere. At the same time, an online communication community will be established to promote interaction and sharing among traditional Chinese sports enthusiasts, and jointly create a positive mental health atmosphere. With collective support and encouragement, more people will be able to maintain their mental health and embrace a more positive and optimistic attitude towards life.

The use of traditional Chinese sports to prevent mental health problems during special periods requires multi-faceted efforts and cooperation. By developing appropriate exercise plans, combining psychological counseling and education, and using Internet technology, we can effectively improve people’s mental health and jointly cope with the challenges brought about by this special period.

### 4.4 Limitations and advantages

Systematic reviews and meta-analysis articles may have certain limitations. First, the scale used to measure mental health in the included literature was inconsistent, leading to greater heterogeneity. Secondly, all the studies were randomized controlled trials and did not use blind methods, and the results were potentially risky. However, exercise intensity was not included in the analysis of study characteristics, which may also be a source of heterogeneity. Fourthly, the included research includes four kinds of traditional Chinese sports, namely Baduanjin, Wushu, Yijinjing and traditional Chinese fitness exercises, which are representative but lacking in comprehensiveness.

Although this systematic review and meta-analysis has certain limitations, it also has many advantages. First of all, register on the PROSPERO website to define the research methods and exclusion criteria in advance, which will lay a solid foundation for the writing of this paper. Secondly, the literature is searched through four Chinese and foreign databases to ensure the universality of literature sources. Thirdly, PRISMA guidelines were strictly followed during literature screening to ensure that the conditions for inclusion of the literature met the requirements. Fourthly, the quality of the included literature was evaluated to ensure the quality of the literature. Fifth, subgroup analysis was conducted from five aspects of intervention measures, frequency, cycle, age and duration of single intervention to explore the impact of traditional Chinese sports on mental health.

## 5 Conclusion

The results of the study show that TCEs have demonstrated the ability to significantly alleviate symptoms of anxiety and depression in the severe context of the global pandemic. Looking ahead to future research, there are several key questions that need to be addressed urgently to further solidify this finding. The first priority is to adopt more rigorous scientific design, such as the implementation of double-blind or triple-blind randomized controlled trials, to improve the scientific and reliable research of the study. Second, by expanding the sample size and adopting a unified and standardized mental health assessment tool, more detailed and comprehensive data can be collected, which can lay a solid foundation for meta-analysis and ensure the universal applicability and accuracy of the conclusions. In summary, this study not only highlights the unique value and cultural charm of traditional Chinese sports in promoting mental health, but also opens up a new horizon for exploring non-pharmacological psychological interventions, and provides valuable inspiration and direction for optimizing mental health intervention strategies.

## Data availability statement

The raw data supporting the conclusions of this article will be made available by the authors, without undue reservation.

## Author contributions

SZ: Data curation, Methodology, Software, Writing – original draft, Writing – review & editing. ML: Writing – review & editing, Methodology, Supervision. LZ: Data curation, Methodology, Writing – original draft.
