# Supplementary material for: Effects of different Chinese traditional exercises on mental health during the COVID-19 pandemic: a systematic review and meta-analysis
Source: Front Public Health. 2024 Jul 31;12:1420035. doi: 10.3389/fpubh.2024.1420035 (PMC11323395; doi:10.3389/fpubh.2024.1420035)
Supplement: Supplementary file 1 [file Table_1.DOCX]

| Supplementary Table S1. Detailed search strategy in four databases. | |
| --- | --- |
| Database | Search strategy |
| Pubmed | (((((((((("Martial Arts"[Mesh]) OR ((((((((((((Arts, Martial) OR (Judo)) OR (Karate)) OR (Kung Fu)) OR (Gongfu)) OR (Gong Fu)) OR (Fu, Gong)) OR (Tae Kwon Do)) OR (Wushu)) OR (Hap Ki Do)) OR (Aikido)) OR (Jujitsu))) OR ((Ch'i Kung) OR (Qi Gong))) OR ("Qigong"[Mesh])) OR ((((((((((Tai-ji) OR (Tai Chi)) OR (Chi, Tai)) OR (Tai Chi Chuan)) OR (Taiji)) OR (Taijiquan)) OR (T'ai Chi)) OR (Tai Ji Quan)) OR (Ji Quan, Tai)) OR (Quan, Tai Ji))) OR ("Tai Ji"[Mesh])) OR (baduanjin)) OR (liuzijue)) OR (yijinjing)) AND ("COVID-19"[Mesh])) AND (("Mental Health"[Mesh]) OR (((Health, Mental) OR (Mental Hygiene)) OR (Hygiene, Mental))) |
| Web  of Science | 1: COVID 19 (Topic) OR 2019-nCoV Infection (Topic) OR 2019 nCoV Infection (Topic) OR COVID-19 (Topic) OR 2019-nCoV Infections (Topic) OR Infection, 2019-nCoV (Topic) OR SARS-CoV-2 Infection (Topic) OR Infection, SARS-CoV-2 (Topic) OR SARS CoV 2 Infection (Topic) OR SARS-CoV-2 Infections (Topic) OR 2019 Novel Coronavirus Disease(Topic) OR 2019 Novel Coronavirus Infection (Topic) OR 2019-nCoV Disease (Topic) OR 2019 nCoV Disease(Topic) OR 2019-nCoV Diseases (Topic) OR Disease,2019-nCoV (Topic) OR COVID19 (Topic) OR Coronavirus Disease 2019 (Topic) OR Disease 2019, Coronavirus (Topic) OR Coronavirus Disease-19 (Topic) OR Coronavirus Disease 19 (Topic) OR Severe Acute Respiratory Syndrome Coronavirus 2 Infection (Topic) OR COVID-19 Virus Disease (Topic) OR COVID 19 Virus Disease (Topic) OR COVID-19 Virus Diseases (Topic) OR Disease, COVID-19 Virus (Topic) OR Virus Disease, COVID-19 (Topic) OR SARS Coronavirus 2 Infection (Topic) OR COVID-19 Virus Infection (Topic) OR COVID 19 Virus Infection (Topic) OR COVID-19 Virus Infections (Topic) OR Infection, COVID-19 Virus (Topic) OR Virus Infection, COVID-19 (Topic) OR COVID-19 Pandemic (Topic) OR COVID 19 Pandemic (Topic) OR Pandemic, COVID-19 (Topic) OR COVID-19 Pandemics (Topic).  2: Mental health(Topic) OR Health, Mental (Topic) OR Mental Hygiene (Topic) OR Hygiene, Mental.  3: Tai Ji (Topic) OR Tai-ji (Topic) OR Tai Chi (Topic) OR Chi, Tai (Topic) OR Tai Chi Chuan (Topic) OR Taiji (Topic) OR Taijiquan (Topic) OR T'ai Chi (Topic) OR Tai Ji Quan (Topic) OR Ji Quan, Tai (Topic) OR Quan, Tai Ji (Topic) OR Qigong (Topic) OR Ch'i Kung (Topic) OR Qi Gong (Topic) OR Martial Arts (Topic) OR Arts, Martial (Topic) OR Judo (Topic) OR Karate (Topic) OR Kung Fu (Topic) OR Gongfu (Topic) OR Gong Fu (Topic) OR Fu, Gong (Topic) OR Tae Kwon Do (Topic) OR Wushu (Topic) OR Hap Ki Do (Topic) OR Aikido (Topic) OR Jujitsu (Topic) OR baduanjin (Topic) OR liuzijue (Topic) OR yijinjing (Topic).  4: #1 AND #2 AND #3. |
| CNKI | (Theme: Eight Pieces of Brocade + Eight Pieces of Brocade Exercise + Fitness Qigong · Eight pieces of brocade + Tai Chi + Traditional Chinese sports + Five Chickens Play + Martial Arts)AND(Theme: Mental health + Anxiety + Depression)AND(Theme: COVID-19 + COVID-19 + 2019-NCOV +SARS-CoV-2 + CoV-19) |
| Wan Fang | All :(traditional Chinese sports or Tai Chi or Qigong or eight Duan Jin or Five poultry plays or Tai Chi or martial arts or six characters) and all :(mental health or anxiety or depression) and all :(COVID-19 or COVID-19 or COVID-19 epidemic or COVID-19 or SARS-CoV-2 or 2019nCoV) |
